# Spiritual Caregiving and Assessments for America’s Religious ‘Nones’: A Chaplaincy Perspective

**DOI:** 10.1007/s10943-023-01757-z

**Published:** 2023-02-07

**Authors:** Garrett Potts, Sage Hewitt, Monica Moore, Alaina Mui, Barbara Lubrano

**Affiliations:** 1 Department of Religious Studies, The University of South Florida, 4202 E Fowler Ave., Tampa, FL 33620 USA; 2 Morsani College of Medicine, The University of South Florida, Tampa, FL USA; 3 Supportive Care Medicine, Moffitt Cancer Center, Tampa, FL USA

**Keywords:** Religious ‘nones’, Patient-centered care, Empathy, Chaplaincy, Spiritual caregiving

## Abstract

One in four American patients now identify as religiously unaffiliated. This study utilizes thematic analysis to deliver qualitative results from in-depth interviews conducted with five chaplains at a premier cancer research institution in Florida to envision what care for their spiritual dimension should look like in practice. It demonstrates *why* the chaplains interviewed suggested that spiritual caregiving still contributes to their holistic wellbeing, and it suggests *how* spiritual care and assessments may be provided to so-called religious ‘nones’—or those who identify as spiritual but not religious, not religiously affiliated, secular humanist, atheist, agnostic, and so on. We conclude with a novel spirituality assessment for use while serving this patient population.

## Introduction

Over the last two decades, research on religion and health has compounded. Graduate and undergraduate programs now offer courses and even full programs at this intersection, and consultancy agencies have begun working with healthcare organizations to promote religious literacy as a way of enhancing patient-centered care. Moreover, regulatory bodies such as the Joint Commission (2021) emphasize that all patients are entitled to whole-person care, which they understand as an integrated concern for the human body, mind, and spirit. In this way, recognizing that one’s deepest values and beliefs are inextricably linked to one’s religion and/or spirituality requires healthcare practitioners to consider a patient’s distinct religio-cultural background within their regimen of care for the ‘whole person.’ The responsibility to provide informed care that centers around the ethics and values of patients from particular social contexts falls on any healthcare practitioner in a patient-facing environment (The Joint Commission, 2021). At the same time, however, the percentage of Americans who identify as religiously unaffiliated has reached 26%, which marks the highest rate in U.S. history (Yeatts et al., 2021).

Our study comes at a crucial turning point in Western healthcare today since a growing number of individuals now identify as atheist, agnostic, secular humanist, spiritual but not religious, and so on (Watson, 2016). Collectively, these groups have become known as religious ‘nones.’ It should be noted that this category is the byproduct of an increasingly common response to a survey question about religious affiliation or lack thereof. Per Saunders et al. (2020), “‘None’ refers, quite simply, to the checking of a box by a survey respondent—‘none’—in response to the question, ‘What is your present religion, if any?’” (p. 424). There may be many reasons that survey respondents choose to identify in this way. Some reasons indicated by the chaplains interviewed as part of this study include unbelief in a higher power as well as religious marginalization or disenfranchisement. It may also be the case that individuals who possess traditional religious beliefs choose to respond in this way for the sake of personal privacy or because they prefer to identify as spiritual but not religious. Whatever the case may be, patient respondents who denote ‘none’ are identified by healthcare practitioners as non-religious or religiously unaffiliated, so we refer to them as such in what follows.

Despite this patient demographic increasing in size, very little research has been conducted about how to best care for the spiritual dimension of survey respondents who identify as religious ‘nones.’ Part of the reason for this gap in the research has to do with the fact that studies at the intersection of religion and health often lack representation by members with religiously unaffiliated identities. Sometimes it is argued that this patient population does not opt into studies focused on religion or spirituality out of fear that identifying as non-religious might result in judgement or prejudice (Hwang et al., 2011). Indeed, “distrust” of atheists and other religiously unaffiliated identities as “immoral” is common, so it is not surprising that many conceal this part of their identity from others (Yeatts et al., 2021). Whatever one’s orientation toward the spiritual is, however, concealing this position is correlated with higher rates of anxiety and depression (Abbot et al., 2020; Abbott & Mollen, 2018; Brewster et al., 2020; Yeatts et al., 2021).

The lack of research involving religious ‘nones,’ combined with emerging evidence pointing to the psychological need for religiously unaffiliated patients to process and vocalize their values and beliefs, leaves us in need of asking important questions about *why* and *how* care for the spiritual dimension of this patient population ought to be conducted. Toward the end of practically envisioning what care and assessment of religiously unaffiliated patients’ spiritual dimension should look like in practice, this study delivers findings from in-depth interviews conducted with five chaplains who serve this patient population.

While spiritual caregiving is not limited to the practice of chaplaincy but is instead the responsibility of all patient-facing healthcare practitioners, as per the Joint Commission (2021), chaplains are subject matter experts at the intersections of religion and health, so we consult them as part of this study to address matters of spiritual caregiving for religious ‘nones.’ Chaplains are trained to serve the spiritual needs of patients from a variety of religio-cultural backgrounds, including those with no religious faith or affiliation. They work to meet the unique spiritual needs of the individual on a case-by-case basis, catering to the particular normative framework of each patient (Koenig, 2013). For this reason, we argue that the chaplains we interview help us understand *why* spiritual caregiving contributes to the holistic wellbeing of religiously unaffiliated patients and *how* spiritual caregiving as well as spirituality assessments may be provided to religious ‘nones.’

## Methods

### Aim and Method

As previously indicated, this article intends to fill a gap in the literature on religion and health and patient-centered care by considering what an empathetic engagement with the spiritual dimension of those who self-report as religious ‘nones’ entails. This aim informs our methodology, and the evidence derived from our study is based on the responses that we received from chaplains while conducting our qualitative research. Our research utilizes an in-depth interview technique that seeks to organize data on the basis of what has been referred to by Sundler et al. (2019) as “thematic analysis.” Per Sundler et al. (2019), the process of thematic analysis first entails “achieving familiarity with the data,” then “searching for meanings and themes” based on participants’ “lived experiences,” and finally “organizing themes into a meaningful wholeness” (p. 736). In what follows, we describe our data collection and provide additional insights pertaining to how this methodology informed our data analysis.

### Participants and Data Collection

At the time of data collection (2020), five experienced chaplains from The University of South Florida’s prestigious Moffitt Cancer Institute were contacted to participate in a study on a volunteer basis that focused on descriptive survey questions to advance our understanding of *why* and *how* spiritual care for religiously unaffiliated populations ought to take place. One of these chaplains also simultaneously worked at a non-university-affiliated hospital as well as a faith-based hospital during this timeframe. Additional demographic information pertaining to the chaplain respondents is represented in Table 1 below.

**Table 1:** Chaplain demographic characteristics

Characteristic	Frequency (%)
*Gender*	
Male	2 (40)
Female	3 (60)
Other	0 (0)
*Age*	
24–40	1 (20)
41–50	2 (40)
51–60	1 (20)
61–77	1 (20)
*Race/ethnicity*	
White/Caucasian	3 (60)
Black/African-American	1 (20)
Other	1 (20)
*Highest education*	
Undergraduate degree	0 (0)
Graduate degree/MDiv	5 (100)
*Role expects respondent to care for…*	
Spiritual or religious patients	5 (100)
Non-spiritual or non-religious patients	5 (100)
*In current role, the respondent has worked with…*	
Spiritual or religious patients	5 (100)
Non-spiritual or non-religious patients	5 (100)

Each chaplain was individually interviewed once via Microsoft Teams, and responses from the chaplains were recorded for future assessment and coding. The following questions comprised our descriptive survey:

1. How do you understand or define spiritual caregiving?
2. What are the major components of spiritual caregiving?
3. Do spiritual assessments provide vital information that is necessary for practitioners to collect, whether or not a patient is religious?
4. Is spiritual caregiving something that should be offered to all, or only the religious?
5. Some healthcare institutions now have ‘humanist’ chaplains to meet the spiritual needs of patients who identify as non-religious. Do you have any examples of how spiritual caregiving is tailored to this population at your institution?
6. Have you ever provided spiritual care to an individual who identifies as non-religious in a healthcare setting?
7. What do you believe the provision of spiritual care for religiously unaffiliated populations ought to look like?

### Data Analysis

Following the interviews, chaplain responses were assessed via the qualitative method of thematic analysis. Toward this end, we first developed a written transcript of each chaplain interview, which was then read multiple times by all the named authors. Second, all the authors independently developed a list of meanings and themes derived from the five chaplain transcripts. Third, a decision about the final thematic list of reasons *why* and *how* spiritual caregiving should be provided to non-religious populations was reached. Lastly, the authors reviewed the transcripts together, coding them based on the aforementioned themes.

With this methodology in mind, what follows is an analysis of the most central reasons *why* the chaplains interviewed all maintained that spiritual assessments and caregiving are a necessary feature of whole-person care for religious ‘nones.’ Then, in the discussion, we extrapolate on insights provided by chaplains during our interviews to develop a shortlist of ‘how-to’ suggestions for conducting spiritual caregiving and rendering spirituality assessments to patients who make up this population.

## Results

Within this section, we address five key themes corresponding to *why* spiritual caregiving and assessments contribute to the holistic well-being of religious ‘nones,’ as indicated by the chaplains interviewed during this study. A brief summary of the most common themes derived from our conversations with chaplains is provided in Table 2 below, and a further analysis of each theme, as well as supporting quotes from the chaplains, are offered following this table.

**Table 2:** *Why* chaplains recommended spiritual caregiving for non-religious populations

Themes	Descriptions
Spiritual Caregiving is Required and Tradition-Constituted by Nature	Regulatory bodies such as The Joint Commission require that healthcare practitioners take into account the sum-total of patients’ values and preferences via spirituality assessments. All religio-cultural backgrounds are to be respectfully accommodated—without forcing any particular ideology upon patients
Spiritual Caregiving Enhances Patient-Centered Care When Making Medical Decisions	Accrediting healthcare agencies identify spirituality as a ‘universal human dimension’—with implications that often influence medical decisions. Like the *body* and *mind* of patients, the human *spirit* is also said to require attention/care. At the heart of spiritual caregiving is the spirituality assessment, which ought to be conducted at regular intervals while providing care for patients
Spiritual Caregiving Empowers Patient Vulnerability by Increasing Perceptions of Empathetic Care	Spiritual caregiving deepens the relational bond between patients and practitioners by inviting patients to be open about their deepest concerns and questions in life, which, in turn, enhances patient perceptions of empathetic care
Spiritual Caregiving Provides Important Insights Pertaining to Patients’ Relational Well-Being	Spirituality assessments and spiritual caregiving more broadly provide insights about patients’ core community, allowing practitioners to better understand whether patients have others who love, care for, and support them
Spiritual Caregiving Provides Important Insights Pertaining to Patients’ Emotional Well-Being	A key feature of spiritual caregiving in general and spirituality assessments in particular entails the evaluation of the emotional well-being of patients (i.e., whether they have a source of joy and peace in their lives) and what might be inhibiting their flourishing

### Spiritual Caregiving is Required and Tradition-Constituted by Nature

Why is tradition-constituted care for religious ‘nones,’ such as atheists, agnostics, secular humanists, spiritual but not religious patients, and so on necessary? While healthcare practitioners often neglect spiritual caregiving for individuals who identify with one of these perspectives, the chaplains interviewed for this study purport that religious ‘nones’ similarly benefit from conversations that consider the traditions they inhabit and the narratives derived from these traditions.

All the chaplains interviewed described working with patients who possess no belief or uncertainty about belief in a higher power as sharing many of the same universal human concerns about finding hope, meaning, purpose, and ways to cope, which are often broadly associated with care for the human spirit. This growing body of literature makes up the discourse on religion and health. Two of the emerging questions within this literature are *why* and *how* to provide spiritual care for religious ‘nones.’ “Although a lot of chaplains cringe at the word humanist,” said Chaplain 1, “seeing such patients’ deep [spiritual] space” helps them to heal, especially when it entails “connecting with whatever patients’ value system or meaning-making system is.” What is important is helping patients “reach that inner part of themselves, so to be a good chaplain—wherever you are—you must put aside whatever your thoughts and beliefs are and focus on that person you’re with” (Chaplain 2). In this way, the chaplains described spiritual caregiving as a universally mandated requirement to care for “whatever gives patients a sense of meaning and…is essential in that person’s spiritual or day-to-day life” (Chaplain 3). More to the point, the reason that spiritual care and spirituality assessments are so essential for religious ‘nones’ and in fact “should be offered to all patients” is because “it gives you a little more depth into how that person is coping with the crisis that is happening in their life at the moment or how the patient is coping with their illness…So, you don’t necessarily have to be religious to be a recipient of spiritual care” (Chaplain 3).

Chaplains need to operate within pluralistic contexts as spiritual ‘chameleons’ at times, so the job “is not for everybody, and some may be better as pastors or rabbis” (Chaplain 4). “As a chaplain,” your primary responsibility is “to support patients and their families’ search for connectedness, meaning, reconciliation, and peace in ways that are meaningful to them, and that might be different from the ways that I find meaning and connectedness or practice my own faith,” Chaplain 4 stated. Beliefs between practitioners and patients on these matters may very well differ, but in these conversations, chaplains “are speaking human to human” about those questions that “are universal…I mean, conversations about life, death, meaning, and purpose—and even prayer—are fairly universal” (Chaplain 4).

When asked about catering to the specific traditions of non-religious patients, it was routinely emphasized that “spiritual caregiving is about helping all persons find peace within their own spiritual understanding” (Chaplain 5). This means “meeting patients where they’re at…the whole idea is not to push your own understanding or faith” (Chaplain 5). Instead, spiritual caregiving “has to be nonjudgmental. It has to be listening, and this requires meeting them where they are at” (Chaplain 5). All of the chaplains emphasized the need to meet patients where they are by speaking and empathizing with them on the grounds of their tradition-based beliefs and using the non-religious language they most closely identify with, be it secular humanist, atheist, agnostic, or otherwise.

### Spiritual Caregiving Enhances Patient-Centered Care When Making Medical Decisions

The chaplains also argued that a primary component of spiritual caregiving and ‘whole person’ care is the spirituality assessment, which enhances patient-centered care and informs medical decisions that need to be made on behalf of a patient. Per the Joint Commission (2021), this assessment may be conducted by a patient’s nurse, primary caregiver, or chaplain at the following times: when a patient first comes into their care, while conducting health maintenance visits, or whenever the existential concerns of a patient seem to call for it.

Examples of when the timing of a spirituality assessment may become immediately necessary include but are not limited to moments such as the following: when patients receive a troubling diagnosis, when patients express fear of death, when patients believe they are being punished by something or someone, or when patients exhibit a loss of hope and connection in life (Koenig, 2013, p. 80). During such times, spirituality assessments, such as the FICA, CSI-MEMO, and ACP, provide a non-religious patient’s entire medical team with insights into how the patient is coping, what makes his or her life meaningful, what cultural or religious beliefs might impact medical treatment, and what social or communal resources may be available to support the patient’s well-being as they navigate important medical decisions (Koenig, 2013, p. 58). With this in mind, we present findings from our chaplains about how spirituality assessments inform medical decisions for religious ‘nones’.

Findings from our chaplains indicated that patients’ spiritual-existential concerns about where they are in life and what they ultimately believe are as important in what they may elect to do medically speaking as their diagnosis and prognosis. In other words, “if a practitioner has a sense of what makes patients tick, so to speak, they’ll be in a better place to offer the best treatments or the best support” when it comes to “providing the medical care that the patient would like to receive” (Chaplain 2). Toward this end, the spirituality assessment was identified as a primary method for “creating a [holistic] plan of care, so the physical plan of care will also connect with the spiritual care that you need to provide for patients” (Chaplain 3). The goal here entails “trying to find the points and spaces in a person’s life where there is a sense of meaning and belonging and purpose,” and how answers to these questions inform patients’ course of treatment (Chaplain 3). With this in mind, we can understand why Chaplain 1 maintained that conducting the spirituality assessment equips practitioners “to be an advocate” for patients. It allows practitioners to be “direct without being abrupt, saying the things you know need to be said” as practitioners and patients work together to make decisions about how to navigate important medical needs based on what patients view as best for their lives as ‘whole persons.’

Perhaps not surprisingly, our chaplains indicated that non-religious patients, much like self-reportedly religious ones, possess a paradigm for understanding themselves in connection to the wider world. In other words, non-religious patients desire “a way of being connected to something that is outside of themselves” (Chaplain 4). Spirituality assessments provide a way of understanding what this paradigm is so that chaplains and other care providers can help the patient see a “glimpse of something outside themselves” that gives their life meaning and purpose (Chaplain 4). When practitioners fail to engage with this paradigm, patients run the risk of “not getting answers in a way they can take them in” (Chaplain 5). Worse, they may shut down for fear of being judged as non-religious. Appropriately understanding and accepting non-religious patients’ views about ultimate reality helps them cope and make difficult decisions with moral and spiritual clarity, thereby allowing them to not feel so “overwhelmed” (Chaplain 5). Spiritual caregiving joins patients and medical practitioners who become empathetic dialogue partners on these matters.

### Spiritual Caregiving Empowers Patient Vulnerability by Increasing Perceptions of Empathetic Care

It goes without saying that being a patient can be an incredibly vulnerable experience due to facing fears surrounding mortality and the loss of control over circumstances. Findings from our chaplains indicate why spiritual caregiving creates an opportunity to enhance holistic care for patients as they face these concerns. It encourages them to be vulnerable in healthy ways that strengthen the patient-provider relationship, promoting perceptions of empathetic care. With these goals in mind, spiritual caregiving was described as holding “a space” for patients to talk about deeply personal concerns “that they’re not going to tell anyone else” (Chaplain 1). This may not always seem necessary. In some cases, for example, patients may be outwardly happy, but Chaplain 1 described a situation where a patient would cry “every time,” despite everyone else on the patient’s care team believing the patient was doing better emotionally. Spiritual caregiving and assessments can break through the surface of patients’ emotional lives to help them heal at deeper levels, Chaplain 1 argued.

Engaging with the spiritual domain gets to the depths of what is coming up for a non-religious patient, and it encourages them not to suppress certain feelings or emotions for fear of judgement. The chaplains all believed that spiritual caregiving is essential for understanding patient needs and how they feel cared for as ‘whole persons.’ One example given was a non-religious patient who disclosed a history of sexual abuse amidst a spirituality assessment. As Chaplain 1 maintained, “that’s information the care team needs to know.” Spiritual caregiving and assessments allow patients to talk about such matters that are troubling them and to do so in a “safe place” that is free of judgment—a place “where they feel heard” (Chaplain 2). During this time, what is most important is that spiritual caregivers are “taking time to listen…without judgment” (Chaplain 3). All the chaplains indicated that spiritual caregiving allows practitioners to address the complex questions about life that we often do not wish to talk about, thereby creating an opportunity to help patients find some sense of solace amidst their vulnerability and concerns.

But how are we to approach these important conversations? When talking with patients, it is necessary to ask questions in such a way that they will “understand their world and their situation better through their own eyes” (Chaplain 4). Such vulnerable conversation allows patients to “self-discover through the process,” giving them “a greater sense of peace” (Chaplain 4).

It must also be emphasized that spiritual caregiving and vulnerable conversations break down when they are treated like one more ‘to-do’ or box to check off for the day. Spirituality assessment guides and checklists like FICA, CSI-MEMO, and ACP can be helpful for thinking about the kinds of questions that may impact medical treatment or help a patient open up and feel cared for, but as Chaplain 4 said, one should not be overly forceful about something “patients are not speaking” about, and practitioners should not push too hard into areas that are not organically “coming up in conversation.” Rather, what is most important is “being able to listen and reflect with the person” and helping them to “make sense of what’s going on in their daily life,” following whatever winding road the conversation takes without making it seem to the patient that you are trying to work through a script (Chaplain 5). Grappling with life’s biggest questions, such as “why do bad things happen to people?” is a key part of spiritual care, and active listening is an important skill for validating a patient’s emotions, such as grief and anger about the bad things that have happened (Chaplain 5). “I truly believe that people just need to be heard,” whether or not someone is religious, Chaplain 5 said, and as a vulnerable conversation partner, spiritual caregivers are there to help “let them work through it themselves.” When spiritual conversations and assessments are not rushed or treated like a script that healthcare practitioners simply must get through—when they are allowed to happen organically between two people talking human to human—then perceptions of having received empathetic care flourish (Koenig, 2013, pp. 96–113).

### Spiritual Caregiving Provides Important Insights Pertaining to Patients’ Relational Well-Being

Findings from our chaplains further indicated that, in order to provide patients with whole-person care, it is necessary for providers to look beyond the individual in front of them to also consider the community with whom they interact. Understanding a patient’s key relationships helps chaplains and other practitioners identify who their primary advocates are in and beyond the healthcare context. This is important because patients’ advocates “help them remember or interpret or understand everything that's being said” about their diagnosis, prognosis, treatment options, and so on (Chaplain 1).

Also important to mention is that, for religious ‘nones,’ ways of relating to the broader world and especially their community represent that which is often taken to be the most significant aspect of their personal spirituality. Thus, spiritual caregivers “don’t need to lead with religion” in order to find what “grounds” patients and contributes to their sense of well-being (Chaplain 1). Rather, spiritual caregivers can gauge how key relationships help non-religious patients to “understand something outside themselves,” thereby allowing them to experience the joy and connection of self-transcendence through meaningful relationships with others in the world, or possibly with something else—like nature (Chaplain 5).

When asked about best practices for gauging non-religious patients’ relational well-being, the chaplains again reinforced the need for spirituality assessments. For example, Chaplain 3 spoke about the ability for spirituality assessments to identify “what those [relational] resources are that the patient is using to cope.” Likewise, Chaplain 2 emphasized the need for identifying these resources so, after discharge, “they have places to go, things to do, and people to contact that strengthen their resilience.” This information helps all healthcare providers “create a physical plan of care” that “will connect with the spiritual plan” as well as their psychological plan, to ensure that the ‘whole’ patient is being considered (Chaplain 2).

Some of the chaplains shared stories of relational tragedy and loss incurred by their patients, which the patients themselves had not previously shared with any other healthcare practitioners. In many cases, this was because no one else had inquired via delivering a spirituality assessment that engaged with such matters. As a way of stressing how significant discussions about the loss of key relationships can be for non-religious patients, Chaplain 4 presented the story of an individual who had “no framework to feel connected to his wife” after she passed and therefore “[felt] crushed and completely alone.” Providing spiritual caregiving in such instances involves helping a patient identify the important connections they still have in the world and promoting “reconciliation through a sense of personal meaning” so that they may have a greater “sense of peace when [they or a loved one are] facing death” (Chaplain 4). Simply creating a space for conversation about non-religious patients’ relational well-being, as all the chaplains argued, is a necessary step to the promotion of personal peace and relational healing. Unfortunately, however, we discovered that it is one step that is frequently overlooked by healthcare practitioners while working with patients who self-report as religious ‘nones.’

### Spiritual Caregiving Provides Important Insights Pertaining to Patients’ Emotional Well-Being

Assessing and caring for patients’ emotional well-being is something that is fundamental to spirituality assessments and spiritual caregiving more broadly. Despite most chaplains and other healthcare professionals lack of formal training in a therapeutic context, they are still expected by the Joint Commission (2021) to help guide patients to a better understanding of their emotional life “in ways that help them gain some resilience” and find “strength” (Chaplain 2). This component of care for the human spirit is vital because “everyday living is not for the faint of heart. There is so much that happens within each day…and if a person possesses an internal strength of spirit—however, they define that—they’re going to do better” (Chaplain 2). Still, however, “there is a fine line between spiritual caregiving and therapy” (Chaplain 1).

Many of the chaplains emphasized that assessing and caring for patients’ emotional well-being is closely tied to active listening and keeping the focus on the patient. “You have to take yourself out of the equation and see the patient’s life from a first-person perspective” (Chaplain 3). Toward this end, Chaplain 4 suggested practicing active listening to promote the emotional well-being of patients by not setting a rigid agenda for meetings but rather “going in with a blank sheet.” “Sometimes I just sit with them and listen to what they’re saying and maybe help them tease out or put words to, or labels on, that which they’re feeling and can’t define,” he said. On some days, the goal of engagement with a patient’s emotional life may be to “highlight” what is coming up for them, and other times the goal is to “affirm or maybe even guide,” but—every day—spiritual caregivers must never lose sight of the fact that space needs to be held for “patients to express themselves,” because “that in itself is spiritual care” (Chaplain 3).

Many of the concerns that impact religiously unaffiliated patients’ emotional lives were described as “universal human concerns” that the field of healthcare normally categorizes as spiritual (Chaplain 4). All of the chaplains spoke with conviction about the need for religiously unaffiliated patients to have a space to voice these concerns because, as Chaplain 5 stated, “we [all] can just get so stuck within ourselves…and that’s not healthy.” Some of the most common concerns that have a bearing on patients’ emotional state—causing them to get ‘stuck’—include “patients’ sense of self-worth…how and where they find meaning in life, or a meaning for their own life, what or whom they feel connected to or part of, their sense of peace or harmony in the world and amidst the events in which they find themselves,” and so on (Chaplain 5). It is interesting to note that these very features were also considered to be “the major components of spiritual caregiving” and spirituality assessments (Chaplain 4). Thus, the universality of these so-called spiritual concerns is what all of the chaplains who were interviewed during this study to maintain that spirituality assessments and the practice of spiritual caregiving should be universal despite one’s religious or non-religious identity.

## Discussion

Considering our chaplains’ argument about *why* spiritual caregiving and spirituality assessments are important for non-religious populations, the primary goal of this discussion section is to provide a spirituality assessment that is more inclusive of the many various identities that those who self-report as religious ‘nones’ may possess. While most of the spirituality assessments on offer entail normative frameworks that imply certain values or beliefs pertaining to the existence of God (usually the God of the Abrahamic traditions) and one’s involvement with distinctly religious communities, suggestions from our chaplains enabled us to develop questions that are more fitting for non-religious populations while still accomplishing the primary goals associated with spirituality assessments, as indicated by the Joint Commission (2021).

In this discussion, we address five key suggestions pertaining to *how* spiritual caregiving and spirituality assessments ought to be delivered to this patient population, and we contextualize our suggestions alongside additional quotes from the chaplains interviewed for further justification. A summary of each ‘how-to’ suggestion is provided in Table 3 below along with a corresponding question for assessment that may be introduced into the flow of conversation while providing spiritual care to non-religious patients. Further discussion around each suggestion and assessment question then ensues before study limitations are highlighted and chapter conclusions are drawn.

**Table 3:** Suggestions and questions for delivering spiritual care to religious ‘nones’

How to deliver spiritual care to religious ‘nones’	Associated spirituality assessment questions
Engage with patients’ normative framework	Do you think your values and beliefs may impact the medical care that you seek? If so, in what way?
Identify patients’ source(s) of transcendent connection	How is your life connected to people and other significant things beyond yourself?
Address trauma holistically	Are you experiencing any degree of anger, despair, or distress because of something that you did or something you witnessed/experienced that betrayed what you believe to be morally right?
Inquire about spiritual practices	Do you use any practices to encourage any form of spiritual growth or personal centeredness? If so, how can we assist you? If not, would you like to consider some strategies that might help you during this time?
Assist with conflict resolution	Are there any relationships for which you desire forgiveness or reconciliation? If so, how can we assist you?

### How Spirituality Assessments Ought to Engage with Patients’ Normative Framework

As indicated within our results section, all the chaplains we interviewed suggested that every patient—religious or otherwise—possesses a paradigm for making sense of questions about the meaning and purpose of human life. “Everybody has a spiritual core and what you want to call that core—whether it's your [non-religious] value system or your Methodist tradition or your Jewish tradition—I don’t care what you call it. Spiritual care is getting in touch with…your deep space,” Chaplain 1 said. Likewise, it was indicated that “if you can understand where a person is coming from [spiritually], then all the other things—you know, the physical, emotional, mental, relational—will make sense,” because patients’ spiritual values and beliefs “hold everything together” (Chaplain 5).

However, several of our chaplains also indicated that it is common for non-religious patients to become victims of the assumption that their lack of faith in a higher power means they do not possess moral values and beliefs. This generalization must be avoided, argued Chaplain 3, because it prevents patients from disclosing whatever the normative framework is that makes up the deep space that holds everything together for them. In place of such stereotypes, research indicates that the best way healthcare practitioners can empathetically engage with their self-reportedly non-religious patients is by actively listening to the values and beliefs that they in fact do voice. Providing a space for religious ‘nones’ to make claims about what they believe and how they understand their purpose “is associated with less psychological distress and higher psychological well-being” by comparison to non-religious patients who conceal their normative framework for fear of judgment (Abbot et al., 2020; Abbott & Mollen, 2018; Brewster et al., 2020; Yeatts et al., 2021).

How then are we to practically engage with non-religious patients’ normative framework so that it may be incorporated into their medical regimen of care and enhance their well-being? One chaplain described the approach taken by her team as follows: “We try to find what the points and the spaces are in that person's life where there is a sense of meaning and belonging and purpose. So, you don't necessarily have to talk about said religious phrases like, you know, God, spirits, or so on…but rather you can talk using spiritual language as to what it is that you know you are here for, what it is that really helps you to move forward” (Chaplain 3). By holding these conversations, healthcare practitioners create a space for non-religious patients to consider how their life is connected to a particular normative framework that guides their quest for meaning and purpose and informs their view of the life worth living, which, in turn, impacts the treatment they will seek for themselves or their loved ones at various stages of the human life cycle, such as birth, sickness, and dying.

With this first ‘how-to’ suggestion in mind, we offer the following spirituality assessment question as a way of grappling with non-religious patients’ normative framework: *Do you think your values and beliefs may impact the medical care that you seek? If so, in what way?*

### How Spirituality Assessments Ought to Identify Patients’ Transcendent Connection(s)

While non-religious populations often remain skeptical about a higher power, it is still important to recognize and appreciate the ways that all patients desire to understand their life as connected to something bigger than themselves (i.e., nature, family and friends, an important cause in the workplace, and so on). Toward this end, spirituality assessments must gauge the degree of connection that patients’ feel to other significant people and things. This corresponds to the Joint Commission’s (2021) suggestion that spiritual caregiving ought to address patients’ relational and causal well-being.

We summarize this ‘how-to’ suggestion as involving the identification of patients’ source(s) of ‘transcendent connection’ because, as the 20th-century psychologist Viktor Frankl (2006) indicated, human flourishing fundamentally requires patients to feel connected to—and even willing to suffer for—important causes and relationships that tether them to something bigger than themselves. Rather than achieving happiness and success by egoistic means, Frankl (2006) argues that recalling those important causes and relationships that patients have pursued as noble ends in themselves helps to crowd in their spiritual sense of flourishing amidst the suffering they presently endure.

Chaplain 1 described “joining” as a “big word” for understanding the practice of spiritual caregiving, allowing us to see more clearly how Frankl’s account of Logotherapy and its emphasis on self-transcendence aligns with this primary goal. Chaplains and others providing spiritual care must promote a “joining presence,” helping others to see how their lives are intertwined with a broader horizon of significance (Chaplain 1). Embodying this kind of presence, practitioners can offer patients “a greater sense of peace,” by gently “helping them discover that they did touch people, and that they did make a difference in the world” (Chaplain 4).

Keeping in mind the vision of self-transcendence espoused by Frankl, coupled with this notion of spiritual caregiving as facilitating a ‘joining presence,’ we offer the following spirituality assessment question for helping patients understand how their lives are tethered to self-transcendent ends: *How is your life connected to people and other significant things beyond yourself?*

### How Spirituality Assessments Ought to Address Trauma Holistically

The chaplains we interviewed also spoke openly about how conducting spiritual assessments revealed that many of their patients had experienced trauma that was inhibiting their well-being. For example, Chaplain 1 reflected on stories about trauma stemming from patients’ former religious communities, which often resulted in a shift in self-reporting about personal religiosity. “Unfortunately, the church has hurt a lot of people, so another non-religious category from atheist [that I’ve worked with] would just be the marginalized and disenfranchised,” he said. Similarly, Chaplain 5 recalled that she has “seen so many people who are angry largely because of organized religion.” The chaplains indicated that patients who have experienced some form of religious trauma may still believe in God. But faith in many cases had become negatively conceptualized or even lost because of traumatic experiences of leaders betraying what patients believed to be right, and this caused a great deal of anger, pain, and despair.

The chaplains also recalled their responsibility to engage with trauma stemming from a sense of personal and moral betrayal that patients had experienced outside of religious communities. After all, such trauma can arise within virtually any social or institutional context where failures in leadership can happen, and spirituality assessments, which engage with patients’ deeply held values and beliefs, often bring this trauma to the surface as patients struggle to reconcile what has happened with what they value or believe. One example from outside a religious institutional context involved a case of sexual abuse that was vocalized during a spirituality assessment that Chaplain 1 conducted with an atheist patient. Traumatic experiences like sexual abuse can fundamentally alter a patient’s beliefs about God, the world, and other people. Thus, in addition to fear-based PTSD symptoms that this patient experienced, which Chaplain 1 believed was the professional responsibility of a qualified therapist to address, there was also some spiritual work to do to help the patient move through a distinctly moral kind of trauma.

How then are chaplains and other spiritual caregivers to engage with patients who have endured traumatic experiences such as the one discussed above, and what exactly is the nature of the spiritual work to be done in these cases? In all the aforementioned cases of trauma, it is striking to note the moral and spiritual wounds that arose during conversations with patients. Hodgson and Carey (2017) associate this common sense of “betrayal” with the condition of “moral injury,” and they describe this trauma as “an existential-ontological wound [inflicted by the self or others] that can have lasting psychological, biological, spiritual, behavioral, and social consequences that chaplaincy/pastoral care practitioners are well placed to assist alongside other health care providers to provide rehabilitation that is holistic” (p. 1224). Initial symptoms may include “(a) shame, (b) guilt, (c) a loss of trust in self, others, and/or transcendent beings” as well as a “(d) spiritual/existential conflict” of meaning, and this may be followed by worsening conditions, such as “(a) depression, (b) anxiety, (c) anger, (d) re-experiencing the moral conflict, (e) social problems…(f) relationship issues…and ultimately (g) self-harm” (Carey & Hodgson, 2018, p. 2).

While Hodgson and Carey (2017) note that moral injury “is a complex phenomenon that requires a holistic approach beyond any one discipline,” they rightly note that spiritual caregivers “can potentially assist with addressing moral injury” (pp. 1223–1224). So, after spiritual caregivers have first understood the nature of the trauma that has occurred, if that trauma entails a possible moral injury to the patient, the second step is to offer a space where these negative experiences may be safely processed for them to “move forward in their own spiritual understanding” and heal emotionally (Chaplain 5).^1^

Considering how spiritual caregiving must navigate trauma to remove spiritual obstacles for patients that will otherwise inhibit their holistic well-being, we propose the following assessment question for conducting this work and determining whether a possible moral injury has occurred: *Are you experiencing any degree of anger, despair, or distress because of something that you did or something you witnessed/experienced that betrayed what you believe to be morally right?*

### How Spirituality Assessments Ought to Grapple with Patients’ Personal Spiritual Practices

It may come as a surprise to some that non-religious patients still very often participate in activities commonly characterized as ‘spiritual practices.’ Such practices may include things like meditation, prayer, and counting one’s blessings (i.e., keeping a gratitude journal). Over the last few decades, thousands of empirical studies have demonstrated how spiritual practices contribute to the holistic well-being of patients, resulting in greater mental, physical, and spiritual resilience. For example, one of the most recent studies, highlighted in the *Journal of Religion and Health,* demonstrated how the practice of “keeping a spiritual diary” was shown to foster spiritual growth while enhancing psychological well-being (Kim et al., 2021).

Insofar as spiritual practices such as the ones mentioned above serve the well-being of religious and non-religious patients alike, chaplains and other healthcare practitioners are encouraged to promote them (but not push them) by expressing a willingness to provide adequate space, time, and any other resources that will be necessary to conduct them without disruption. This may require spiritual caregivers to do some scheduling work on behalf of the patient to ensure that there is a time of day set aside for spiritual practices, just like times are scheduled each week for physical therapy and other activities that are integral to a patient’s holistic well-being.

Because practices associated with the spiritual growth of non-religious patients may vary widely, we suggest the following open-ended question for gauging how caregivers may support them in their activities: *Do you use any practices to encourage any form of spiritual growth or personal centeredness? If so, how can we assist you? If not, would you like to consider some strategies that might help you during this time?*

### How Spirituality Assessments Ought to Assist with Conflict Resolution

The last ‘how-to’ suggestion offered as part of this discussion centers around conflict resolution. Conflict resolution entails the process by which two or more people settle a dispute. Successful resolutions often rely on important spiritual themes, such as grace, forgiveness, reconciliation, and so on. As Chaplain 2 indicated, “even in non-religious groups there are ways that people are taught how to make amends to themselves and others and to seek forgiveness for actions and things like that. So, even a person who is not religious can benefit” [from this aspect of spiritual caregiving].

Chaplain 4 provided two of the most common examples of conflict resolution that surface for non-religious patients, and it is worth noting that the need for conflict resolution becomes particularly prominent in both cases when patients are faced with the reality of their mortality. The first story involved a non-religious patient who was dying and not at peace because of a “ruptured family relationship that had been weighing on him for 30 years.” Only after conducting a spirituality assessment did the existential weight of this relational conflict surface in conversation. The chaplain was then able to reconnect this man with his estranged family member of 30 years, and that family member ultimately visited him in the hospital. The impact that this had on the patient’s emotional state at the end of his life was profound. After the reconciliation, “he had a sense of peace about facing death” (Chaplain 4).

The second example of conflict resolution that sometimes becomes necessary for subsets of the non-religious population (such as the spiritual but not religious) involves reconciliation with God. “Many patients are angry with God because of something that happened in their lives, and they may need help to figure it out—or to seek reconciliation” (Chaplain 4). Examples may include sudden losses or hardships that have surprised patients earlier in their lives, such as the loss of a child or a spouse or a wrongful conviction. So, it is important to emphasize that, despite a patient identifying as non-religious, they may still exhibit troubling emotions directed toward a divine one which spiritual caregiving and assessments can help them to process.

With these examples mind, the final spirituality assessment question that we offer as part of this research is the following: *Are there any relationships for which you desire forgiveness or reconciliation? If so, how can we assist you?*

## Limitations

There are a few noteworthy limitations of this research that cannot go unmentioned. The first involves the small sample size of chaplains we interviewed as part of this study. Given the labor-intensive nature of the methodology that informed our qualitative approach, we chose to interview only five chaplains, all of whom have several years of experience working with both religious and non-religious populations. Future research on this subject would benefit from expanding the number of qualitative interviews conducted with chaplains possessing similar levels of experience.

A second limitation of this study has to do with the homogeneous context wherein all these chaplains presently serve. While chaplains interviewed as part of this study had prior experience working with other religiously and non-religiously affiliated healthcare institutions across various geographical contexts within the United States, all of them presently served at a secular cancer research institution in Florida during the time that interviews were conducted. That being said, future research on this subject would benefit from interviewing chaplains who work in a greater variety of geographical and institutional contexts at the present time of the study.

## Conclusion

With the limitations of this study in mind, we nonetheless maintain that the objectives we set out to achieve were accomplished. Conversations with the chaplains who were interviewed as part of this research mark some of the earliest practitioner-focused qualitative data that addresses *why* and *how* spiritual caregiving and spirituality assessments ought to be conducted while working with religious ‘nones.’ Given that the percentage of patients who identify as non-religious is rising with each new generation in America (Yeatts et al., 2021), this is an important gap to fill within the literature.

We hope that the research offered here will influence healthcare workers’ delivery of a more patient-centered approach to providing care for non-religious populations. Care for the spiritual dimension of non-religious populations, so our chaplains argued, still requires at least some degree of person-centered background knowledge that may be acquired via a spirituality assessment, which grapples with questions about patients’ traditional affiliation or aversion to certain religious affiliations, their sense of hope, meaning, purpose, and connection in life, as well as how their distinct values and beliefs may impact their treatment paradigm. Toward this end, we encourage those who conduct spiritual caregiving to consider using the spirituality assessment that we designed when they are serving religious ‘nones.’ This assessment includes five key questions as summarized in Table 4.

**Table 4:** Associated Spirituality Assessment Questions

1. Do you think your values and beliefs may impact the medical care that you seek? If so, in what way?
2. How is your life connected to people and other significant things beyond yourself?
3. Are you experiencing any degree of anger, despair, or distress because of something that you did or something you witnessed/experienced that betrayed what you believe to be morally right?
4. Do you use any practices to encourage any form of spiritual growth or personal centeredness? If so, how can we assist you? If not, would you like to consider some strategies that might help you during this time?
5. Are there any relationships for which you desire forgiveness or reconciliation? If so, how can we assist you?

## Footnotes

1 While it is unfortunately beyond the scope of this article to address what the provision of this space for processing moral trauma ought to look like, Carey and Hodgson (2018) have developed a technique based on the practice of “Pastoral Narrative Disclosure” (PND) for chaplains and others trained in spiritual care. It aligns nicely with the kind of intervention that may be necessary based on patients’ response to the assessment question that we developed within this section.
